# Fucoidan Extracts Ameliorate Acute Colitis

**DOI:** 10.1371/journal.pone.0128453

**Published:** 2015-06-17

**Authors:** Qi Ying Lean, Rajaraman D. Eri, J. Helen Fitton, Rahul P. Patel, Nuri Gueven

**Affiliations:** 1 Pharmacy, School of Medicine, University of Tasmania, Hobart, Tasmania, Australia; 2 University of Technology MARA, Puncak Alam, Selangor, Malaysia; 3 School of Health Sciences, University of Tasmania, Launceston, Tasmania, Australia; 4 Marinova Pty Ltd, Cambridge, Tasmania, Australia; Charité-Universitätsmedizin Berlin, GERMANY

## Abstract

Inflammatory bowel diseases (IBD), such as ulcerative colitis and Crohn’s disease, are an important cause of morbidity and impact significantly on quality of life. Overall, current treatments do not sustain a long-term clinical remission and are associated with adverse effects, which highlight the need for new treatment options. Fucoidans are complex sulphated, fucose-rich polysaccharides, found in edible brown algae and are described as having multiple bioactivities including potent anti-inflammatory effects. Therefore, the therapeutic potential of two different fucoidan preparations, fucoidan-polyphenol complex (Maritech *Synergy*) and depyrogenated fucoidan (DPF) was evaluated in the dextran sulphate sodium (DSS) mouse model of acute colitis. Mice were treated once daily over 7 days with fucoidans via oral (*Synergy* or DPF) or intraperitoneal administration (DPF). Signs and severity of colitis were monitored daily before colons and spleens were collected for macroscopic evaluation, cytokine measurements and histology. Orally administered *Synergy* and DPF, but not intraperitoneal DPF treatment, significantly ameliorated symptoms of colitis based on retention of body weight, as well as reduced diarrhoea and faecal blood loss, compared to the untreated colitis group. Colon and spleen weight in mice treated with oral fucoidan was also significantly lower, indicating reduced inflammation and oedema. Histological examination of untreated colitis mice confirmed a massive loss of crypt architecture and goblet cells, infiltration of immune cells and oedema, while all aspects of this pathology were alleviated by oral fucoidan. Importantly, in this model, the macroscopic changes induced by oral fucoidan correlated significantly with substantially decreased production of at least 15 pro-inflammatory cytokines by the colon tissue. Overall, oral fucoidan preparations significantly reduce the inflammatory pathology associated with DSS-induced colitis and could therefore represent a novel nutraceutical option for the management of IBD.

## Introduction

Inflammatory bowel disease (IBD) is a known medical burden in most developed countries and a significant cause of morbidity [1]. Due to the nature of chronic inflammation involving the gut mucosa, patients present with symptoms such as abdominal pain, diarrhoea, bloody stool and weight loss, which significantly decreases their quality of life [2, 3]. Currently, pharmacological and surgical interventions are the two main management approaches for IBD. Drugs such as corticosteroids, aminosalicylates and immune-suppressants, which aim to decrease inflammation, show limited effectiveness for long term remission and are associated with significant side effects [4]. Monoclonal antibodies that inhibit tumour necrosis factor (TNF-α) such as infliximab have shown clinical usefulness but at a relatively high cost [5, 6]. Furthermore, about 20–40% of patients do not respond to TNF-α inhibitors, while in up to 50% of patients, the therapeutic response is lost after 1–2 years [7–9]. Surgery is reserved for about 20–30% of patients who are unresponsive to medication and develop life-threatening complications such as perforation, refractory rectal bleeding and toxic megacolon [2, 3]. Even after surgery, patients are predisposed to the risk of postoperative complications such as bowel obstruction, anastomotic strictures, pouchitis, sexual dysfunction and pouch failure [2, 3]. Therefore, there is an urgent need for new treatment options that are safe, able to sustain clinical remission and improve mucosal gut healing.

Animal models have become a useful tool to study the pathophysiology of IBD and to test the *in vivo* efficacy of potential therapeutic agents [10]. A number of chemically-induced colitis models have been described that use different colitogenic substances, such as dextran sulphate sodium (DSS), di- or tri-nitrobenzene sulfonic acid, oxazolone or acetic acid [11]. DSS-induced colitis in mice is the most commonly used model; it is associated with severe epithelial damage and a robust inflammatory response in the colon [12, 13]. DSS-treated mice show signs of acute colitis including rectal bleeding, body weight loss, passage of fresh blood through the anus, usually in or with stools and diarrhoea [12, 14]. Macroscopic and histological examination reveals shortening of the intestine, submucosal ulceration and loss of globlet cell and crypt structure, as well as infiltration of large numbers of inflammatory cells into the colonic mucosa [12, 14, 15]. These macroscopic and histopathological changes are associated with the excessive production of pro-inflammatory cytokines [16, 17]. Although this animal model has limitation regarding the accurate representation of the clinical course of IBD, it nevertheless recapitulates the common clinical features of inflammation and histopathology seen in human disease [12]. Importantly, despite its shortcomings, this model has been validated through the use of several clinically used therapeutic agents against IBD [17].

Given the need for new treatment options for IBD, we have tested two fucoidan extracts in the DSS-induced colitis model. Fucoidans are a class of sulphated, fucose-rich polymers found in edible brown macroalgae and echinoderms, which are commercially available as dietary supplements. Fucoidans exhibit different bioactivities, which have been reported in many pre-clinical *in vitro* and *in vivo* models [18]. However, the efficacy and characteristics of these bioactivities can vary significantly and depend on the source, species, molecular weight, composition and structure of the molecules, as well as on the route of administration. The reported bioactivities of fucoidans are diverse and include anticoagulation, blocking of lymphocyte adhesion and invasion, inhibition of multiple enzymes, induction of apoptosis, antiviral activity and, most importantly for this study, a substantial anti-inflammatory activity [18–20]. Specifically, current evidence that fucoidans can be beneficial for regulating gut health is derived from a variety of pre-clinical models and studies. In a mouse model of chronic colitis orally delivered fucoidan (from *Cladosiphon*) was reported to downregulate the levels of the pro-inflammatory cytokine IL-6, which was also confirmed through *in vitro* studies [21]. Similarly, in a rat model of acute colitis, an orally delivered polysaccharide food supplement containing fucoidan reduced monocyte numbers and improved clinical markers of colitis [22]. Fucoidan is also known to protect against aspirin-induced gastric ulcers in a rat model [23] and is known from pre-clinical and clinical investigations to inhibit gastric pathogens [24–26].

Fucoidan extracts are commercially available as dietary supplements and for this study two preparations of fucoidan from *Fucus vesiculosus* were compared. One is a highly purified preparation of fucoidan (depyrogenated fucoidan, DPF) with a relatively low molecular weight, whereas the other (Maritech *Synergy*) is a fucoidan that is naturally complexed with polyphenols. We demonstrate here for the first time that orally delivered fucoidan or a fucoidan polyphenol complex both significantly suppress the inflammatory response in the acute DSS-induced model of colitis. These findings could open the way towards new treatment options for patients with IBD.

## Materials and Methods

### Animal colitis model

All animal experiments were approved by the Committee of Animal Ethics of the University of Tasmania (A13576) and were conducted in accordance with the Australian Code of Practice for Care and Use of Animals for Scientific Purposes (8^th^ Edition 2013). Male C57BL/6 mice (aged 8–10 weeks; 21–27 g, average weight ≈ 25 g) were obtained from the University of Tasmania animal breeding facility and housed in a temperature-controlled, non-sterile environment under a 12-hour day/night light cycle. Mice derived from different litters and at different time points over a period of three weeks were randomized into the different treatment arms based on body weight and age to ascertain comparable group compositions. Body weights of mice were assessed daily over an initial acclimation period of one week. All mice were non-fasting and had access to food and drinking water (autoclaved tap water) *ad libitum*. Colitis was induced by supplementing 3% w/v of dextran sulphate sodium (DSS, MW = 40,000–50,000, USB, Affymetrix Inc, Ohio, USA) in the drinking water of mice from day 1 to day 8 (day of termination). Control mice received drinking water without DSS.

### Formulation of fucoidan extracts

Depyrogenated fucoidan (DPF) and fucoidan-polyphenol complex (Maritech *Synergy*), extracts (Table 1) from marine brown seaweed *Fucus vesiculosus* (bladder wrack) were provided by Marinova Pty Ltd, Tasmania, Australia. DPF injection solution (5 mg / ml) was prepared by dissolving DPF in water for injection and filtered using a 0.45 μm filter. Fucoidan-containing food mash was prepared as described previously [27]. Briefly, 0.75 g of fucoidan extract (DPF or *Synergy*) and 9 g of sucrose (4% w/w of food mash) were separately well-suspended/ dissolved in autoclaved water before they were homogenously mixed into a solution with a final weight of 105 g. Food powder (120 g) was then dispersed slowly into the mixture with stirring to make a food paste. The food mash was aliquoted as 3 g/dish and stored at -20°C.

**Table 1:** Fucoidan extract composition.*

Extract	Neutral Carbohydrates (%)	Sulphates (%)	Counter-ions (%)	Poly-phenols (%)	Uronic acids (%)	Peak MW (kDa)
**Fucus-polyphenol** (Maritech *Synergy*)	40.2	21.8	6.3	26.2	3.6	203.1
**High-purity fucoidan** (DPF)	59.5	26.6	11.9	< 0.5	1.4	61.7

*Data provided by Marinova Pty Ltd

Both extracts are polydisperse, containing fractions from 5 kDa to above 1000 kDa. Fucus-polyphenol is the more polydisperse and features a greater proportion of high molecular weight molecules. The major component of the neutral carbohydrate content of both compounds is fucose. The fucus-polyphenol extract (Maritech *Synergy*) contains 25.8% fucose overall, whilst the high-purity fucoidan (DPF) contains 53.3% fucose.

### Treatment with fucoidan extracts

Healthy control (HC) mice received standard chow while another group which received DSS in their drinking water and only vehicle through the food represented the disease control (DSS). Three treatment groups (n = 10 mice / group), where all mice received DSS in their drinking water, were randomized for treatment with fucoidan extracts for 7 days (day 1—day 7). The first treatment group was injected intraperitoneally with DPF injection solution (IPDPF, 0.25 mg / mouse, 10 mg / kg / day). The second and third treatment groups were given oral DPF and Maritech *Synergy* respectively (ODPF and OS; 10 mg / mouse, 400 mg / kg / day). For the oral treatment groups, the mice were single-caged throughout the experiment to ascertain the defined daily intake of fucoidan from prepared food mash. In addition, all mice had access to normal food pellets *ad libitum*.

### Evaluation of intestinal inflammation

Mice were weighed and observed daily for stool consistency and the presence of blood in the stool and sign of gross bleeding on the anus site [11]. Stool samples were obtained from individual mice and were tested by using Hemocult II slides (Beckman Coulter Inc., California, USA).

### Termination of experiment and tissue sampling

Mice were sacrificed on day 8 by carbon dioxide inhalation followed by cervical dislocation. Upon dissection, the spleen was removed and weighed. The full colon was then carefully removed. Colon length was measured between the ceco-colic junction and the proximal rectum. Subsequently, the entire colon was opened longitudinally to remove the faecal content before the wet weight of each colon was measured. The colon was then divided longitudinally to be used for either fixation in 10% v / v buffered formalin and histological analysis or for tissue explant culture.

### Histologic evaluation of colitis

Fixed colons were processed into paraffin blocks and were cut into 4 μm sections, which were stained with hematoxylin and eosin. Subsequently, all sections were graded in an investigator-blinded manner regarding the severity of the tissue damage using light microscopy (Leica DM2500, Leica Microsystems Pty Ltd, NSW, Australia and Leica application suite version 3 software) at 100× / 400× magnification. The severity of colitis was assessed according to the criteria described previously [28–30].

### Tissue explant culture and measurement of cytokine levels

Tissue from the distal colon was cut and washed with cold (PBS) before each sample was transferred into a 12 well-plate containing 1 ml / well of Roswell Park Memorial Institute (RPMI) 1640 culture medium (In Vitro technologies Pty Ltd, Victoria, Australia) supplemented with 10% v/v foetal calf serum (FCS, Gibco, Life Technologies, Victoria, Australia), penicillin (100 mU / L) and streptomycin (100 mg / L) (Sigma-Aldrich Pty Ltd, NSW, Australia). After 24 hours of incubation, supernatants were collected and stored at -80°C until further analysis. A Bio-Plex Pro Mouse cytokine 23-plex kit (Bio-Rad Laboratories, Inc., California, USA) was used to determine the cytokine levels in the tissue culture supernatants. Following the manufacturer instructions, cytokine standards and undiluted culture supernatants were tested in duplicate on a Bio-Plex 200 instrument equipped with Bioplex Manager software, version 6 (Bio-Rad Laboratories, Inc., California, USA). The cytokine levels were normalized by dividing the cytokine results (pg / ml) by the measured tissue weight (mg) and expressed as pg cytokine / ml / 10 mg of tissue.

### Statistical analysis

All statistical analyses were performed using GraphPad Prism (version 6, GraphPad Software Inc, CA, USA). Statistical significance was evaluated using one or two way analysis of variance (ANOVA), followed by a multiple comparison test: Dunnett’s test or Tukey’s test. Pearson’s correlation coefficient (r^*2*^) was determined for the relationship between two variables when necessary. A *p* value of < 0.05 was considered statistically significant.

## Results

### Oral fucoidan extracts ameliorate colitis-induced weight loss

**Fig 1:**
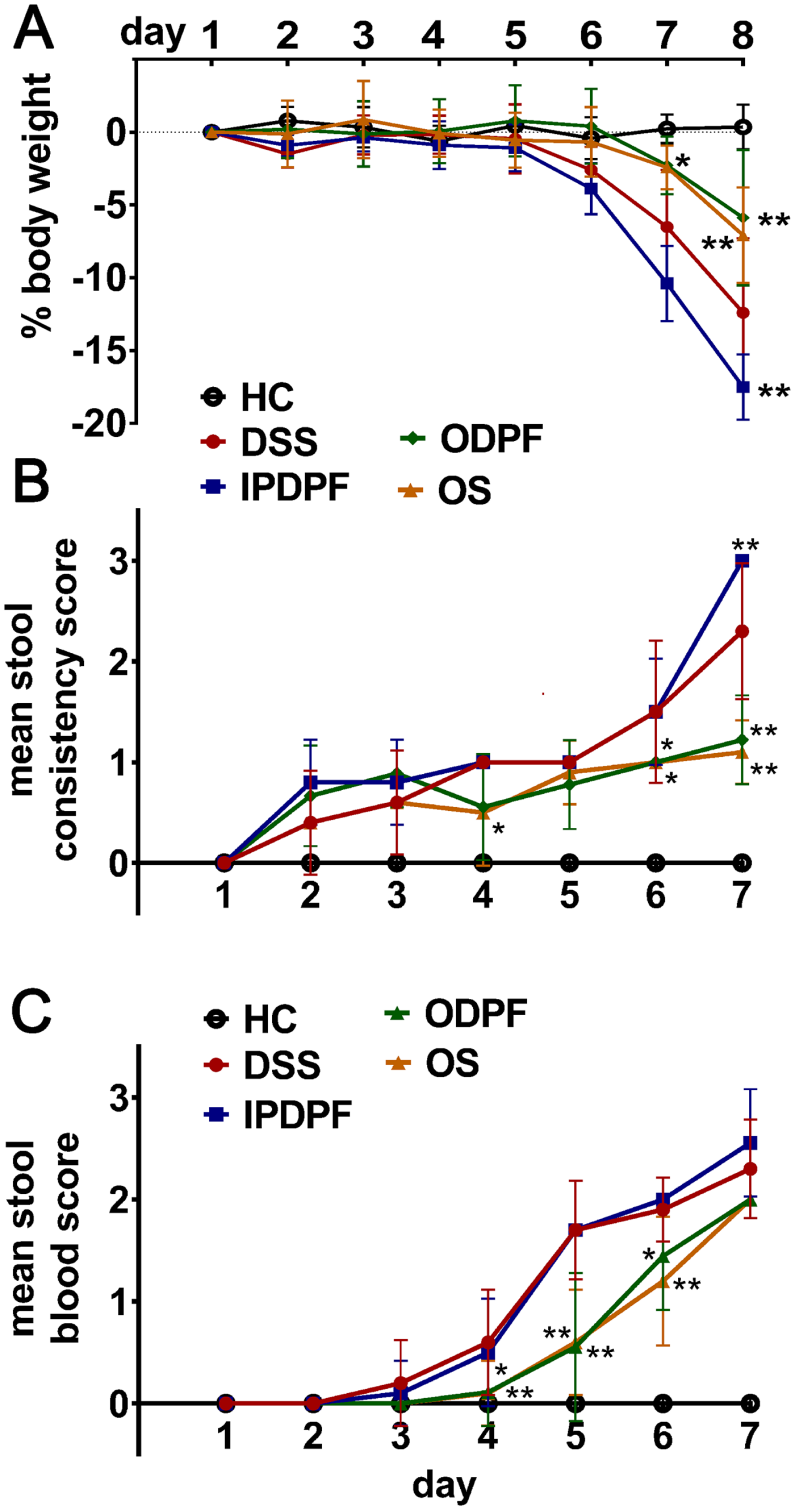
Effect of fucoidan extracts during acute colitis. (A) Daily changes of body weight during colitis induction in C57BL/6 mice with and without fucoidan extracts treatment versus healthy control. Body weight changes, expressed as percentage, were calculated by dividing body weight on each day with the initial body weight before the start of DSS treatment. Stool samples were scored for (B) consistency and (C) occult blood on a daily basis during experiment. Data represent percentage or mean ± SD of n = 6–10 animals. Significance is indicated by *p < 0.05 and **p < 0.01 using two-way ANOVA followed by Tukey’s post-test. Healthy control (HC); untreated colitis (DSS); intraperitoneal injection of depyrogenated fucoidan (IPDPF); oral treatment of depyrogenated fucoidan (ODPF); oral treatment of Maritech *Synergy* (OS).

Mice receiving DSS in the drinking water developed acute colitis, evidenced by a significant reduction of body weight, on day 8 (-12.3 ± 4.3%; *p* < 0.0001) (Fig 1A). Oral administration of both fucoidan extracts significantly reduced body weight loss compared to untreated colitis (Fig 1A). Statistically significant differences were obtained on day 7 (*p* = 0.05) and day 8 (*p* < 0.0001), with a maximum weight loss of only 5.9 ± 4.4% for mice treated with oral DPF (ODPF). Likewise, mice treated with oral *Synergy* (OS) only showed a weight loss of 7.8 ± 3.1% on day 8 which was significantly different to untreated mice (*p* = 0.0005). There was no significant difference between the effectiveness of the oral fucoidan extracts. In contrast, intraperitoneal (i.p) DPF (IPDPF) showed no protection but resulted in significantly increased weight loss on day 8 (-17.5 ± 2.1%, *p* = 0.001) compared to the untreated mice.

### Oral fucoidan extracts delay the development of diarrhoea and the appearance of blood in stool

After exposure to DSS, untreated mice showed clear changes in stool consistency (Fig 1B) and the presence of blood in stool (Fig 1C) compared to mice without exposure to DSS. As early as day 2, mice receiving DSS developed soft stool that still contained formed pellets. On day 3, stool of untreated mice with DSS tested positive for occult blood. The severity of symptoms progressively increased towards the end of the experiment, when mice had bloody soft stool or diarrhoea. Consistent with retention of body weight, mice which received oral DPF (ODPF) or *Synergy* (OS) showed significant protection against diarrhoea over the entire observation period (Fig 1B). Oral fucoidan extracts also significantly protected against the presence of blood in stool from day 4, although this protection was lost at day 7 (Fig 1C). Intraperitoneal DPF (IPDPF) was not effective in alleviating DSS-induced blood in stool (Fig 1C) or worsening of stool consistency (Fig 1B).

### Oral fucoidan extracts protect against changes to colon and spleen

**Fig 2:**
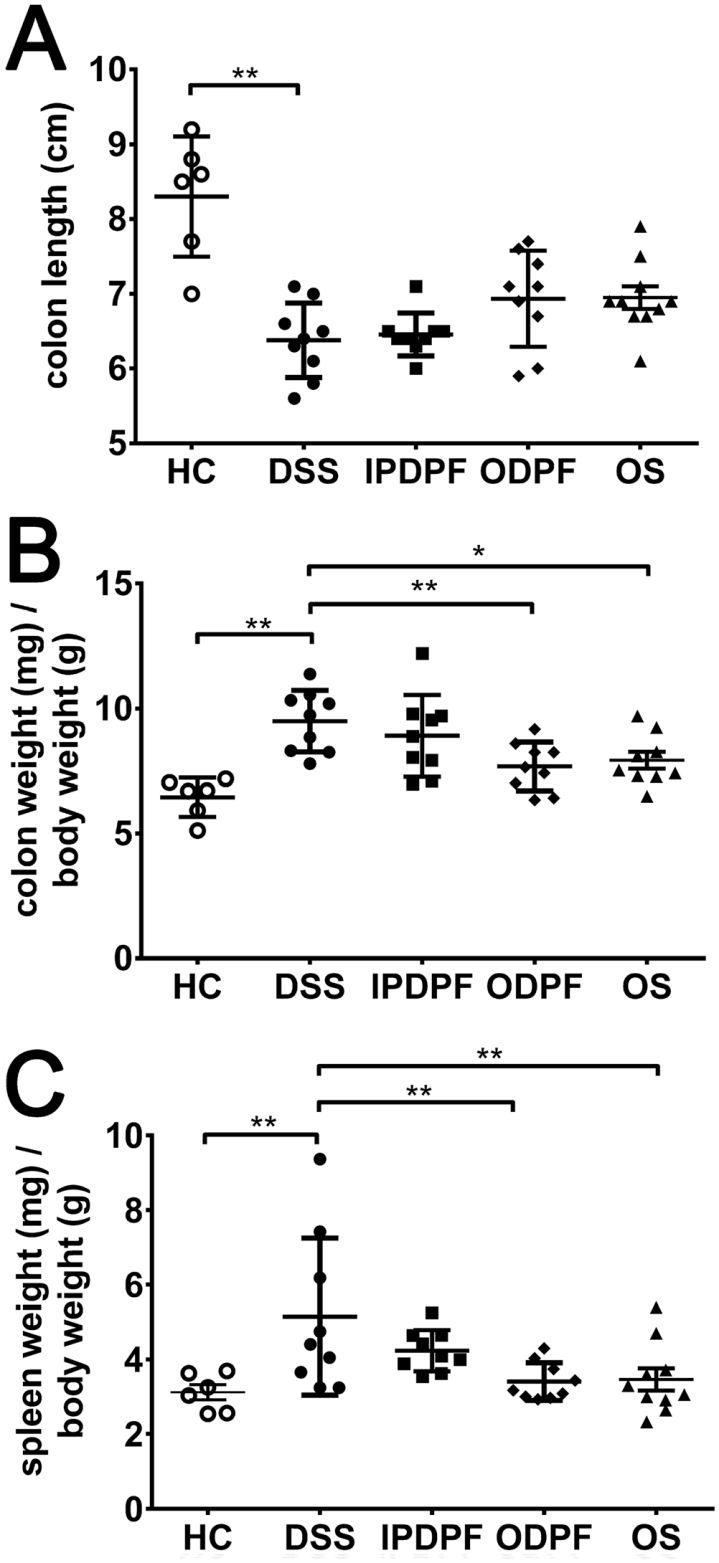
Effect of fucoidan extracts on colon and spleen. Colons were measured for their length (A) and weight. Spleens were also removed and weighed. The organ weights were then presented as a ratio of colon weight (B) or spleen weight (C) over body weight respectively. Data represent the mean ± SD of n = 6–10 animals. Significance is indicated by *p < 0.05 and **p < 0.01 using one-way ANOVA followed by Dunnett’s post-test. Healthy control (HC); untreated colitis (DSS); intraperitoneal injection of depyrogenated fucoidan (IPDPF); oral treatment of depyrogenated fucoidan (ODPF); oral treatment of Maritech *Synergy* (OS).

After the observation period, DSS-induced changes to colon and spleen were analyzed. DSS-treated mice showed significantly shortened colon length (6.4 ± 0.5 cm; about 77%) compared to healthy control mice (8.3 ± 0.7 cm) (Fig 2A). Although protection by oral fucoidan did not reach significance compared to untreated mice, there was a trend towards reduced colon shortening for ODPF and OS (6.9 ± 0.6 cm and 7.0 ± 0.5 cm; respectively; about 84% of healthy controls). Wet colon weight, an indicator of intestinal oedema and inflammation, was presented as the ratio of colon weight over body weight (mg / g). As expected, the untreated colitis group showed the highest relative weight (9.4 ± 1.2 mg / g) (Fig 2B). The relative colon weight was significantly reduced by 19.1% for ODPF (7.7 ± 0.9 mg / g; *p* = 0.01) and 16.5% for OS (7.9 ± 1.0 mg / g; *p* = 0.03) compared to the untreated group (Fig 2B). Consistent with the previous results, IPDPF showed no effect with regards to colon length (6.5 ± 0.3 cm) (Fig 2A) or relative colon weight (8.9 ± 1.5 mg / g) (Fig 2B).

Intestinal inflammation is associated with spleen enlargement [13] and as expected, the relative spleen weight of the mice receiving DSS in our study was significantly increased (5.1 ± 2.0 mg / g; *p* = 0.006) compared to spleens from healthy control mice (n = 6). Oral administration of either DPF or *Synergy* protected against the increased relative spleen weight (3.4 ± 0.5 mg / g and 3.4 ± 0.9 mg / g, respectively; *p* = 0.009) compared to untreated colitis mice, whereas IPDPF did not affect relative spleen weight (4.2 ± 0.5 mg / g, *p* = 0.28).

### Oral fucoidan extracts decrease colon damage and infiltration of inflammatory cells

**Fig 3:**
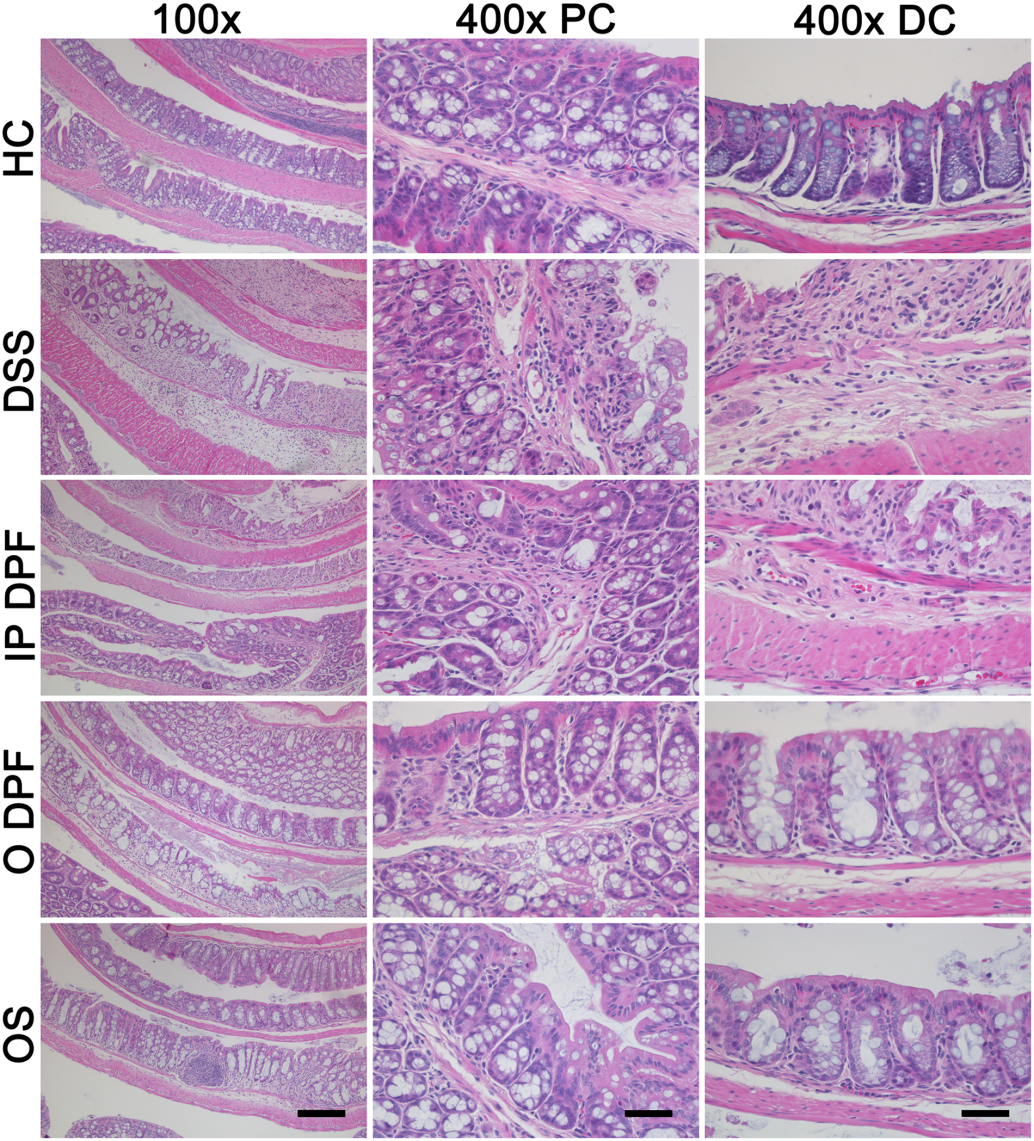
Effect of fucoidan extracts on colon histology. Representative hematoxylin and eosin stained colon sections of healthy controls, untreated mice with colitis and colitic mice that received fucoidan extracts. Scale bars = 100 μm for 400× and 400 μm for 100× magnification. Healthy control (HC); untreated colitis (DSS); intraperitoneal injection of depyrogenated fucoidan (IPDPF); oral treatment of depyrogenated fucoidan (ODPF); oral treatment of Maritech *Synergy* (OS).

**Fig 4:**
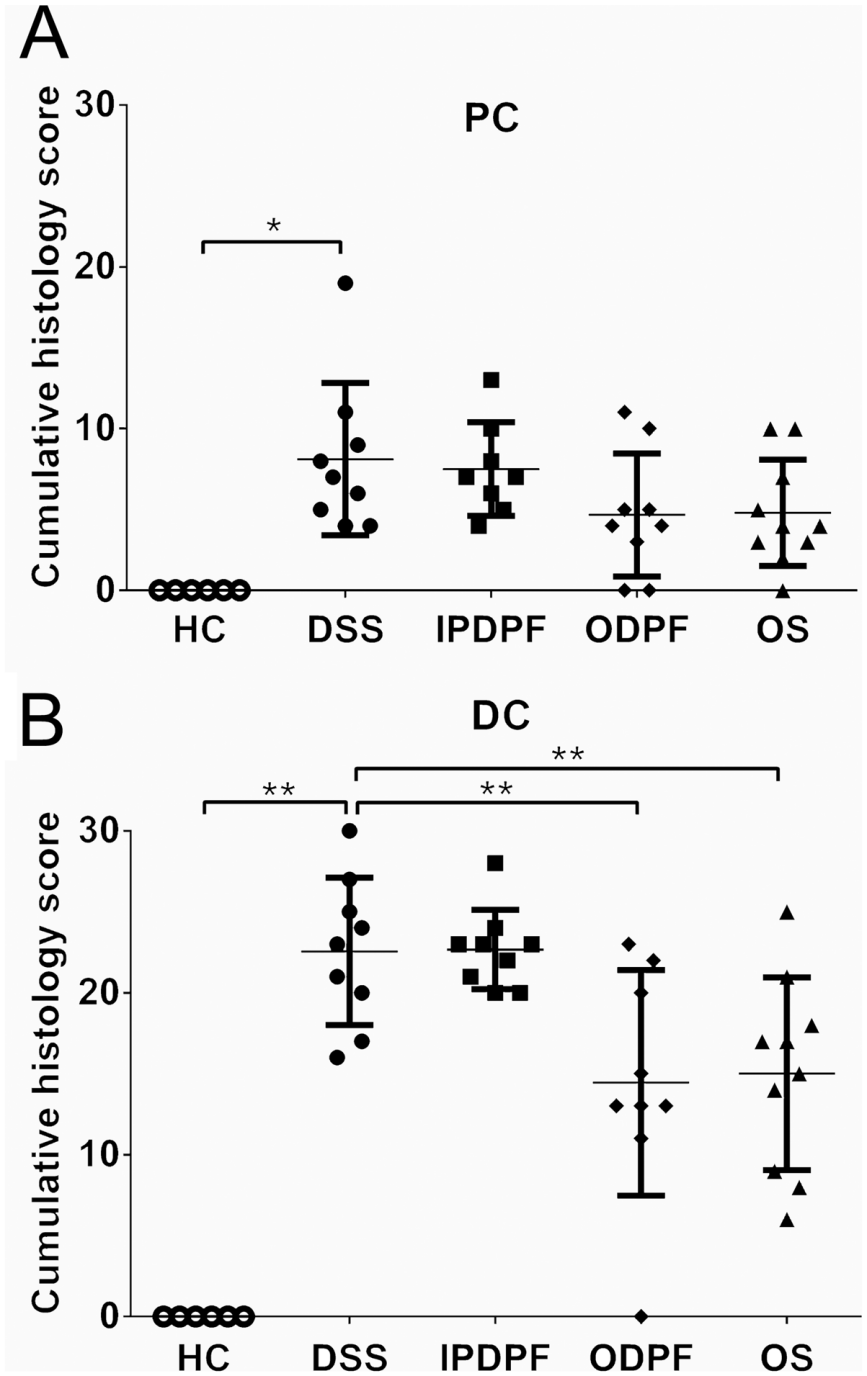
Effect of fucoidan extracts on colon tissue. Cumulative histology damage scores for (A) proximal colon (PC) and (B) distal colon (DC). Data represent the mean ± SD of n = 6–10 animals. Significance is indicated by *p < 0.05 and **p < 0.01 using one-way ANOVA followed by Dunnett’s post-test. Healthy control (HC); untreated colitis (DSS); intraperitoneal injection of depyrogenated fucoidan (IPDPF); oral treatment of depyrogenated fucoidan (ODPF); oral treatment of Maritech *Synergy* (OS).

Histological examination of colon tissue revealed that all DSS-treated mice displayed erosion or destruction of epithelium, crypt distortion, loss of goblet cells, submucosal edema, increased colonic wall thickness and inflammatory cellular infiltration in the colon, mostly affecting the distal colon (Fig 3). While healthy mice showed no signs of histological colon damage (score 0), DSS resulted in cumulative damage scores of 8.1 ± 4.4 for the proximal colon (Fig 4A) and 22.6 ± 4.3 for distal colon (Fig 4B). Co-treatment with oral fucoidan extracts induced protection as evidenced by retention of colonic structure, reduced infiltration of inflammatory cells and reduced submucosa edema (Fig 3), which overall resulted in a significant overall reduction of cumulative histological disease scores for the distal colon (14.4 ± 6.6; 36.3%, *p* = 0.005 and 15.0 ± 5.6; 33.6%, *p* = 0.007 for ODPF and OS respectively) (Fig 4B). In contrast histology scores for the proximal colon demonstrated no significant protection by oral fucoidan extracts. IPDPF also showed no marked protection against distal or proximal colon damage, as indicated by high histological scores for the distal colon (22.7 ± 2.3) (Fig 4B).

### Oral fucoidan extracts reduce the levels of pro-inflammatory cytokines

**Fig 5:**
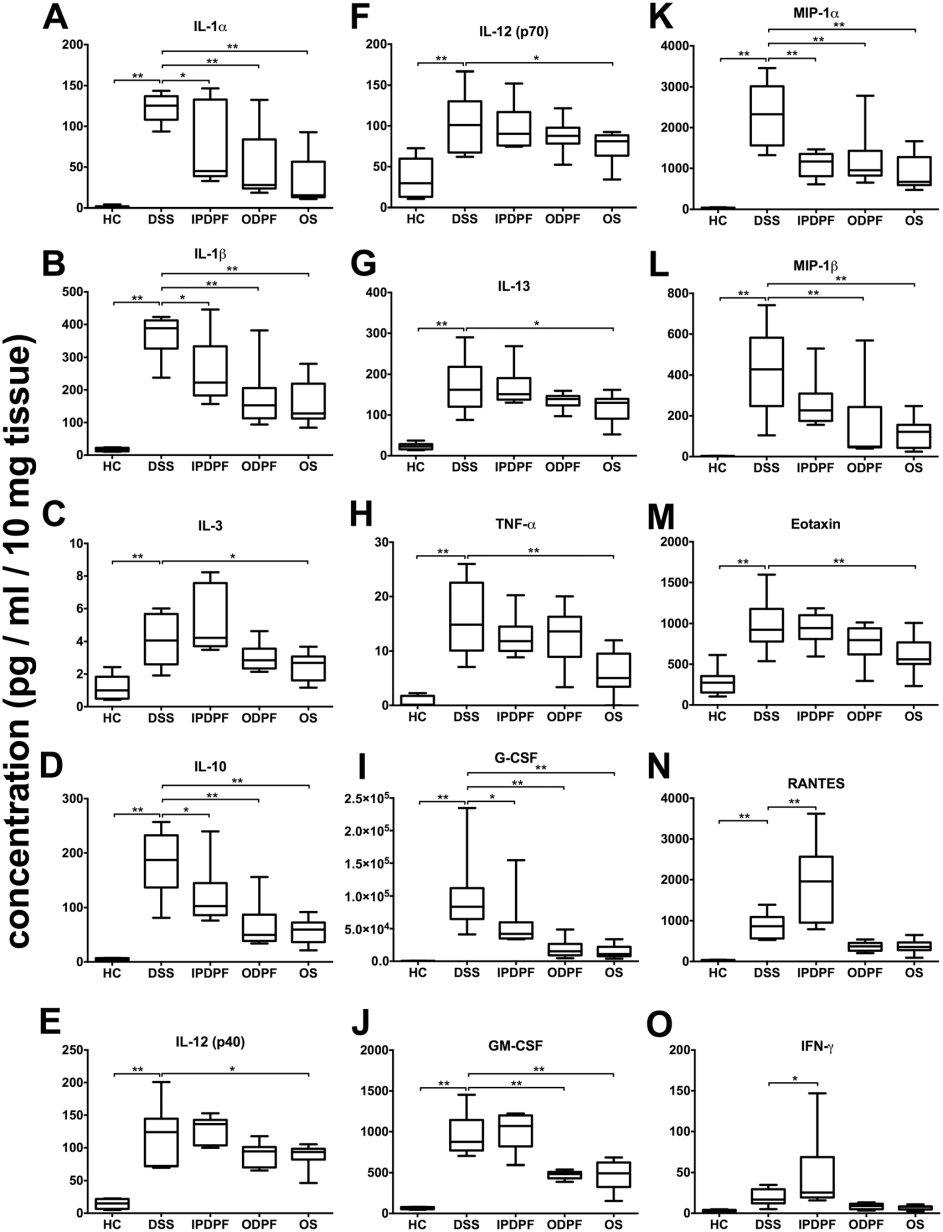
Effect of fucoidan extracts on colon-derived cytokine levels. Distal colon tissue samples were cultured for 24 hours. The supernatants were assessed for cytokine levels using a Bio-Plex assay kit. Cytokine levels in the supernatant were normalized to tissue weight to obtain pg / ml of cytokines/ 10 mg of tissue. Data represent minimum, 25th percentile, median, mean, 75 percentile and maximum of cytokine levels of n = 5 animals. Significance is indicated by *p < 0.05 and **p < 0.01 using one-way ANOVA followed by Dunnett’s post-test. Interleukin (IL); tumor necrosis factor-α (TNF-α); granulocyte colony-stimulating factor (G-CSF); granulocyte-macrophage colony-stimulating factor (GM-CSF); macrophage inflammatory protein (MIP); regulated and normal T cells expressed and secreted (RANTES); interferon-γ (IFN-γ); healthy control (open circle); untreated colitis (closed circle); intraperitoneal injection of depyrogenated fucoidan (closed square); oral treatment of depyrogenated fucoidan (closed diamond); oral treatment of Maritech *Synergy* (closed triangle).

**Fig 6:**
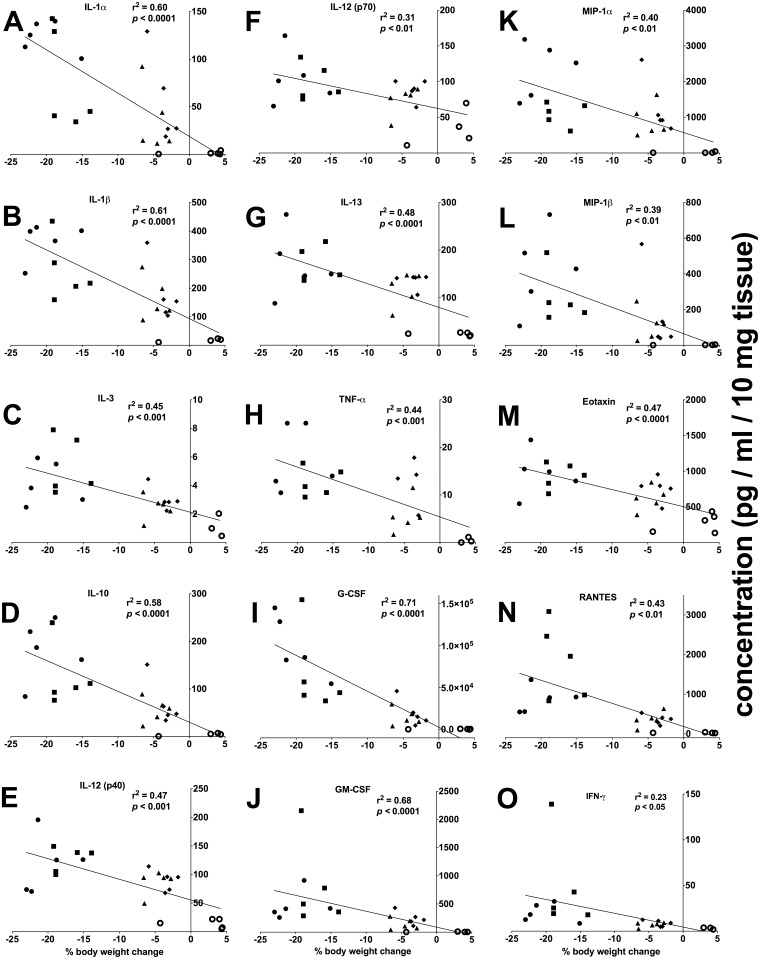
Correlation between colon-derived cytokine levels and treatment-induced changes to body weight. Changes in cytokine levels by fucoidan extracts were correlated with the changes to body weight for individual mice. The value of Pearson correlation coefficient (r^2^) is reported and significance is indicated by *p* value. Interleukin (IL); tumor necrosis factor-α (TNF-α); granulocyte colony-stimulating factor (G-CSF); granulocyte-macrophage colony-stimulating factor (GM-CSF); macrophage inflammatory protein (MIP); regulated and normal T cells expressed and secreted (RANTES); interferon-γ (IFN-γ); healthy control (HC); untreated colitis (DSS); intraperitoneal injection of depyrogenated fucoidan (IPDPF); oral treatment of depyrogenated fucoidan (ODPF); oral treatment of Maritech *Synergy* (OS).

Cytokines are important mediators of the mucosal immune response. We therefore quantified colon tissue-derived cytokine levels to characterize the extent of inflammation during the acute colitis in the DSS model and the effects of fucoidan extracts. DSS treatment induced a significant increase of a multitude of cytokines during the acute colitis period (Fig 5 and S1 Fig). Compared to untreated colitis, both oral fucoidan extracts significantly lowered the levels of IL-1α, IL-1β, IL-10, MIP-1α, MIP-1β, G-CSF and GM-CSF (Fig 5 and Table 2). In addition, oral *Synergy* also significantly reduced the levels of IL-3, IL-12 (p40), IL-12 (p70), IL-13, TNF-α and eotaxin (Fig 5 and Table 2). Importantly, at the level of each individual mouse, the fucoidan-treatment-induced reduction of pro-inflammatory cytokine levels significantly correlated with the protection against body weight loss (Fig 6). For two cytokines, MCP-1 and IL-6, DSS increased their levels beyond the upper detection limit of the assay, while co-treatment with both oral fucoidan extracts decreased their levels back into the measurable range (S1 Fig). In contrast to the oral fucoidan formulations, the overall cytokine response to IPDPF was inconsistent. While IPDPF reduced the levels of IL-1α, IL-1β, IL-10, G-CSF and MIP-1α, the levels of IFN-γ and RANTES were significantly increased by this treatment (Fig 5 and Table 2).

**Table 2:** Effect of fucoidan extracts on colon-derived cytokine levels.

Cytokine	Intraperitoneal DPF	Oral DPF	Oral *Synergy*
	% change (*p* value)	% change (*p* value)	% change (*p* value)
**IL-1α**	-36.4 (0.017)	-55.9 (0.0002)	-71.3 (< 0.0001)
**IL-1β**	-28.6 (0.016)	-51.2 (< 0.0001)	-55.7 (< 0.0001)
**IL-3**	n.s	n.s	-50.7 (0.0242)
**IL-10**	-31.1 (0.0308)	-62.2 (< 0.0001)	-69.2 (< 0.0001)
**IL-12 (p40)**	n.s	n.s	-26.5 (0.0386)
**IL-12 (p70)**	n.s	n.s	-29.4 (0.0398)
**IL-13**	n.s	n.s	-30.8 (0.0179)
**MIP-1α**	-52.8 (< 0.0001)	-46.5 (0.0004)	-60.9 (< 0.0001)
**MIP-1β**	n.s	-60.0 (0.0037)	-72.7 (0.0004)
**G-GSF**	-43.7 (0.0193)	-80.6 (< 0.0001)	-85.2 (< 0.0001)
**GM-GSF**	n.s	-51.0 (< 0.0001)	-51.9 (< 0.0001)
**Eotaxin**	n.s	n.s	-36.6 (0.0041)
**IFN-γ**	85.6 (0.0309)	n.s	n.s
**TNF-α**	n.s	n.s	-68.0 (0.0003)
**RANTES**	115.8 (< 0.0001)	n.s	n.s

Supernatants from colon tissue explant culture were assessed for cytokine levels. The percent (%) change of treatment group versus the DSS colitis group for each cytokines is shown. Not significant (n.s); interleukin (IL); macrophage inflammatory protein (MIP); granulocyte colony-stimulating factor (G-CSF); granulocyte-macrophage colony-stimulating factor (GM-CSF); interferon-γ (IFN-γ); tumor necrosis factor-α (TNF-α); regulated and normal T cells expressed and secreted (RANTES).

## Discussion

We have shown here that oral fucoidan extracts can effectively protect against DSS-induced acute colitis. Not only did the oral formulations alleviate the macroscopic pathologies such as body weight and stool consistency, they also significantly reduced the underlying intestinal inflammation. Although previous reports have demonstrated anti-inflammatory activities of fucoidan and fucoidan-containing extracts in different experimental models *in vitro* and *in vivo* [18, 22, 31–36], this is the first report to demonstrate that dietary fucoidan extracts from *Fucus vesiculosus* are highly effective in ameliorating experimental colitis through a consistent downregulation of a significant number of pro-inflammatory cytokines.

Many different cytokines are involved in the pathogenesis IBD [37]. They form a complex network that regulates mucosal inflammation and affects the integrity of epithelium [38]. Since pro-inflammatory cytokines from patient samples correlate with disease activity [39, 40], treatments that modulate these mediators are likely to be of therapeutic use. In our study, we quantified a larger number of cytokines compared to previous studies to get a broader understanding of the immunological cytokine response during intestinal inflammation and especially in response to fucoidan treatment.

Despite the numerous reports describing the different anti-inflammatory activities of fucoidans, there is no information available that would highlight a single mode of action that could be responsible for the effects observed in this study. It is now generally accepted that the inflammatory response in patients and animal models of IBD is predominantly macrophage driven [41–43]. During intestinal inflammation, monocytes are recruited and differentiated into macrophages within the lamina propria [42] and it is believed that the initial exposure of interstitial macrophages to bacterial antigens is responsible for the activation of macrophages and that at least in the DSS model these activated macrophages subsequently stimulate the proliferation of T cells [44]. In particular the highest DSS-induced cytokines in our study G-CSF and MIP-1α, which were up-regulated by more than 200-fold and 100-fold respectively, support the strong involvement of macrophages in this model. On the other hand, fucoidans were reported to influence pro-inflammatory signalling molecules and pathways in macrophages, such as p38, Erk, JNK [34], HMGB1 and NF-κB [45]. These signalling pathways are essential for macrophages to become a major source of other pro-inflammatory cytokines and consistent with this previously described *in vitro* activity of fucoidans [46], we observed a marked reduction of most measured cytokines that originate from inflamed colon tissues.

Particularly TNF-α is thought to play a significant role in inflammatory cellular signalling, which is reflected by the successful clinical use of TNF-α inhibitors in patients with IBD [5]. Consistent with this hypothesis, macrophage-derived TNF-α production was reportedly blocked by fucoidan treatment in an *in vitro* co-culture model of gut inflammation [35, 47]. However, in contrast to previous observations our results illustrate that the highly purified fucoidan extract (DPF) was unable to normalize the expression of TNF-α, while the polyphenol containing extract (*Synergy*) significantly reduced elevated TNF-α levels, which suggests additional benefits of the polyphenol content of fucoidan extracts to suppress TNF-α signalling.

In addition to macrophages, epithelial cells are also involved in driving the inflammatory cascade by secreting IL-6 in both patients and animal models of colitis [21]. Although our measurements for IL-6 under conditions of acute colitis exceeded the range of the assay, our data nevertheless suggest that both oral fucoidan extracts also reduce IL-6 levels (S1 Fig), which would be consistent with previous observations [21].

One of the first studies of fucoidan use in a mouse model of acute colitis demonstrated reduced mucosal damage and retained crypt architecture [20]. In that study, in contrast to our approach of dietary treatment, fucoidan was administered intravenously. In line with this route of administration and results from *in vivo* microscopy, the authors convincingly argued that the beneficial effects were most likely explained by the well-known inhibition of selectins by fucoidans [18, 48], which can prevent lymphocyte adhesion. In addition, fucoidans have also been reported to inhibit the next stage of lymphocyte tissue infiltration, the invasion, by directly inhibiting the expression [49] and activity of matrix metalloproteases (MMPs) [18, 50]. MMPs are implicated in tissue invasion of immune cells under pro-inflammatory conditions and, consistent with this inhibitory activity of fucoidans, histology results in our colitis model suggested significantly reduced lymphocyte invasion and oedema.

Given the significant size of fucoidans, which is between 5 and 1000 kDa, it appears plausible that the route of administration can significantly alter efficacy due to restricted absorption and tissue distribution of the parent molecule. Since oral fucoidans are likely to reach the target tissue in the case of IBD, most pre-clinical animal studies have therefore preferred oral formulations over intravenous injections for this indication [20–22]. Not only is this the most preferable form of administration for patients with IBD, it is also potentially the least stressful for rodents in pre-clinical *in vivo* studies. It nevertheless has to be stated that oral administration by gavage can also constitute a significant stress to the animals and if not performed accurately can be associated with damage to the GI tract. Consequently, previous studies used fucoidan-containing food chow to treat rodents, without having control over the ingested amount of fucoidan [21]. In contrast, the present study is the first to accurately control the amount of fucoidan in food that has been ingested per day by mixing a defined amount of fucoidan in a separate portion of food chow that the animals eat preferentially before eating their normal food. This method has been successfully used before [27], is very well tolerated and also allows to study the unstressed behaviour of the animals due to minimal animal handling.

In line with previous reports, our results strongly support the use of oral fucoidan extracts while, in contrast, intraperitoneal fucoidan was unable to reduce disease severity in our disease model. In fact, in the case of body weight as a clinical marker, intraperitoneally administered fucoidan appeared to worsen the condition. This result was also reflected at the level of cytokines, where IPDPF showed a trend towards increasing the levels of some pro-inflammatory cytokines in DSS-treated animals. These results are not unexpected, since a recent study indicated that intraperitoneal fucoidan can effectively lead to the activation of dendritic cells (DCs) in the spleen [51]. The activation of DCs in this report was associated with increased production of IL-6, IL-12 and TNF-α. In line with an activation of immune cells by intraperitoneal fucoidan, we observed increased levels of IFN-γ and RANTES and a trend towards increased levels of IL-3, GM-CSF, IL-12(p40), IL-5 and IL-17 by IPDPF. In contrast to the study by Jin *et al* [51], especially oral *Synergy* reduced the production of IL-12 and TNF-α, and likely also of IL-6, by cells of the intestinal tissue. These discrepant bioactivities highlight that the multiple activities of fucoidans are highly dependent on the specific formulation and route of administration used, which determines distribution to specific tissues in the organism. In line with several reports, our results do not provide any evidence for toxicity by oral fucoidan extracts [18, 52, 53].

Overall, our results indicate that oral fucoidan extracts can significantly reduce the pathology associated with acute colitis induced by DSS. Given that multiple factors including disease location, type of inflammation, pathogenic mechanisms and the combination of multiple cytokines affect the response to colitis treatment [38], it is conceivable that drugs that target a single pro-inflammatory cytokine are likely to be limited in their ability to offer an effective maintenance therapy for IBD over extended periods of time. In contrast, fucoidans that simultaneously modulate multiple pro-inflammatory mechanisms and mediators could provide a more sustainable strategy against intestinal inflammation. This study extends our current understanding of oral administration of fucoidan extracts during acute colitis, which is a crucial step towards its use for the treatment of colitis. The ability of fucoidan extracts to reduce inflammation and to retain epithelial integrity along with the possibility of oral delivery serves as justification to develop and evaluate fucoidan extracts as therapeutic alternatives for patients with IBD.

## Supporting Information

S1 Fig Effect of fucoidan extracts on colonic cytokine levels. Distal colon tissues were cultured for 24 hours before supernatants were assessed for cytokine levels using a Bio-Plex assay kit. Cytokine levels were normalized to tissue weight to obtain pg / ml of cytokines / 10 mg of tissue. Data represent minimum, 25th percentile, median, mean, 75 percentile and maximum of cytokine levels of n = 5 animals. Concentrations fell out of range (OOR), exceeding upper detection limits; n.s, non-significant; interleukin (IL); monocyte chemotactic protein-1 (MCP-1); keratinocyte-derived chemokine (KC); healthy control (HC); untreated colitis (DSS); intraperitoneal injection of depyrogenated fucoidan (IPDPF); oral treatment of depyrogenated fucoidan (ODPF); oral treatment of Maritech *Synergy* (OS)
(TIF)
